# Re-interpretation of PAM50 gene expression as quantitative tumor dimensions shows utility for clinical trials: application to prognosis and response to paclitaxel in breast cancer

**DOI:** 10.1007/s10549-018-05097-5

**Published:** 2019-01-23

**Authors:** Nicola J. Camp, Michael J. Madsen, Jesús Herranz, Álvaro Rodríguez-Lescure, Amparo Ruiz, Miguel Martín, Philip S. Bernard

**Affiliations:** 1 Huntsman Cancer Institute, University of Utah, Salt Lake City, USA; 2 Department of Internal Medicine, University of Utah, Salt Lake City, USA; 3 Spanish Breast Cancer Group, GEICAM, Madrid, Spain; 4 Hospital Universitario de Elche, Elche, Spain; 5 Instituto Valenciano de Oncología, Valencia, Spain; 6 Instituto de Investigación Sanitaria Gregorio Marañón, Universidad Complutense, Madrid, Spain; 7 Centro de Investigación Biomédica en Red de Oncología, CIBERONC-ISCIII, Madrid, Spain; 8 Department of Pathology, University of Utah, Salt Lake City, USA

**Keywords:** Gene expression, Multi-gene, Biomarkers, Breast cancer, Dimensions

## Abstract

**Background:**

We recently showed PAM50 gene expression data can be represented by five quantitative, orthogonal, multi-gene breast tumor traits. These novel tumor ‘dimensions’ were superior to categorical intrinsic subtypes for clustering in high-risk breast cancer pedigrees, indicating potential to represent underlying genetic susceptibilities and biological pathways. Here we explore the prognostic and predictive utility of these dimensions in a sub-study of GEICAM/9906, a Phase III randomized prospective clinical trial of paclitaxel in breast cancer.

**Methods:**

Tumor dimensions, PC1–PC5, were calculated using pre-defined coefficients. Univariable and multivariable Cox proportional hazards (PH) models for disease-free survival (DFS) were used to identify associations between quantitative dimensions and prognosis or response to the addition of paclitaxel. Results were illustrated using Kaplan–Meier curves.

**Results:**

Dimensions PC1 and PC5 were associated with DFS (Cox PH *p* = 6.7 $\times$ 10^−7^ and *p* = 0.036), remaining significant after correction for standard clinical–pathological prognostic characteristics. Both dimensions were selected in the optimal multivariable model, together with nodal status and tumor size (Cox PH *p* = 1.4 $\times$ 10^−12^). Interactions with treatment were identified for PC3 and PC4. Response to paclitaxel was restricted to tumors with low PC3 and PC4 (log-rank *p* = 0.0021). Women with tumors high for PC3 or PC4 showed no survival advantage.

**Conclusions:**

Our proof-of-concept application of quantitative dimensions illustrated novel findings and clinical utility beyond standard clinical–pathological characteristics and categorical intrinsic subtypes for prognosis and predicting chemotherapy response. Consideration of expression data as quantitative tumor dimensions offers new potential to identify clinically important patient subsets in clinical trials and advance precision medicine.

**Electronic supplementary material:**

The online version of this article (10.1007/s10549-018-05097-5) contains supplementary material, which is available to authorized users.

## Introduction

Tumor dimensions are orthogonal, quantitative traits derived from expression data in intrinsic gene sets. We previously derived five tumor dimensions for breast cancer as a new representation of tumor diversity using the PAM50 gene set. Quantitative dimensions were superior to categorical intrinsic subtypes for identifying tumor characteristics that clustered in high-risk pedigrees and identified a novel genome-wide significant breast cancer susceptibility locus [1]. The increased power to differentiate tumors from high-risk pedigrees indicates the potential to distinguish important genetic diversity, providing new opportunities for precision genomics. Furthermore, as independent tumor traits, dimensions can be used alone or in combination—a flexible framework for prediction modeling and a new avenue to explore associations and interactions with clinical endpoints.

Breast cancer is heterogeneous in terms of cellular make-up, molecular alterations, response to therapies, and patient outcomes. Gene expression has been shown to classify breast tumors into groups that have similar biology and clinical behavior [2, 3]. Unsupervised hierarchical clustering of “intrinsic” genes [4, 5] has repeatedly found 4 major breast tumor subtypes (Luminal A, Luminal B, HER2-enriched, and Basal-like). These categorical subtypes are robustly identified using the standardized PAM50 gene set [6]. Gene expression signatures, including PAM50 subtypes and its risk of relapse score, have been shown to be prognostic beyond standard clinical–pathological staging for estrogen receptor-positive (ER+) disease in the adjuvant chemotherapy setting [3, 7, 8], but have not been found to be predictive to particular regimens [3]. In estrogen receptor-negative (ER−) breast cancer, there has been evidence that subtypes are predictive for response/resistance to particular chemotherapy regimens. For instance, patients with Basal-like tumors have been shown to not receive additional benefit from an anthracycline-based chemotherapy regimen, whereas the HER2-enriched group has a significant benefit [9].

Standard of care for women with locally advanced breast cancer is to treat with a combined anthracycline-taxane regimen. Despite the additional toxicity of including a taxane (e.g., peripheral neuropathy), metadata analyses from multiple randomized clinical trials have shown it provides a marginal but significant benefit to survival [10–15]. Identifying patients who do not benefit from the addition of a taxane would reduce unnecessary toxicities and allow randomization to more effective treatments.

In the GEICAM Spanish Breast Cancer Group trial 9906 (GEICAM/9906), the PAM50 categorical intrinsic subtypes have previously been shown to be prognostic but not predictive of treatment response, and the PAM50 proliferation score both prognostic and predictive of survival in women given a weekly taxane regimen [16]. Here, we use the same GEICAM/9906 clinical trial data as a case study to investigate the utility of quantitative tumor dimensions to identify associations with prognosis or response to the addition of paclitaxel.

## Methods

### GEICAM/9906 clinical trial

GEICAM/9906 was a prospective adjuvant multicenter randomized Phase III clinical trial (*n* = 1246 women randomized and eligible from November 1, 1999 to June 30, 2002) comparing six cycles of adjuvant fluorouracil, epirubicin, and cyclophosphamide (FEC) (*n* = 632) to four cycles of FEC followed by eight weekly cycles of paclitaxel at 100 mg/m2 (FEC-P, *n* = 614) in node-positive operable breast cancer patients. Patients that were hormone receptor positive (ER and/or PR positive by immunohistochemistry [IHC]) were given adjuvant tamoxifen. The primary endpoint was 5-year disease-free survival (DFS). The study was performed in accordance with the Declaration of Helsinki, approved by the ethics committees at all participating institutions and the Spanish Health Authority, and registered at http://www.clinicaltrials.gov (Identifier Code: NCT00129922). Patients gave written informed consent for therapy randomization and molecular analyses. Further details of the study design, CONSORT trial flow diagram, and patients’ characteristics have been previously reported [13, 17].

Formalin-fixed, paraffin-embedded (FFPE) breast tumor samples, collected at the time of surgery, were gathered and processed in a central laboratory in Alicante as described previously [17]. H&E sections from each FFPE tissue block were reviewed by a pathologist and three 0.6-mm tumor cores extracted from areas containing representative invasive breast carcinoma. Cores were placed in triplicate in tissue microarrays, and sections used for immunohistochemistry (IHC) analysis [ER, progesterone receptor (PR), and Ki-67 status] and chromogenic in situ hybridization (CISH) experiments (HER2 status). Where possible, two or more 1-mm tumor cores were also obtained and sent to the Huntsman Cancer Institute at the University of Utah for RNA extraction and expression profiling using the PAM50 RT-qPCR assay (*n* = 820). These were shown to be from a representative cohort of the larger GEICAM/9906 trial [16, 17].

### Tumor dimensions

Five breast tumor dimensions, PC1–PC5, were previously defined [1] using PAM50 gene expression data from the LACE [18] and Pathways [19] (LACE/PW) population-based breast cancer cohorts. Briefly, a principal components analysis was performed on the PAM50 gene expression matrix from LACE/PW samples. Five dimensions were identified, which explained 68.1% of the total 50-gene expression co-variances (30.5%, 18.9%, 10.2%, 5.3%, 3.2% explained by PC1–PC5, respectively). Each dimension is an independent, quantitative trait, a linear combination of expression across all PAM50 genes (Table S1).

The PAM50 gene expression profiling for LACE/PW and GEICAM/9906 samples was performed in the same laboratory. The pre-analytical (RNA extraction from FFPE punches), analytical (RT-qPCR on the LC480), and post-analytical (reference control and housekeeper normalization) methodologies were identical. We used gene coefficients previously derived from the LACE/PW data [1] (Table S1) to determine the tumor dimensions PC1–PC5 in the GEICAM/9906 samples.

### Clinical–pathological characteristics and PAM50 intrinsic subtypes

The following clinical–pathological variables were considered, with categorization pre-specified to match previous GEICAM/9906 studies [13, 16, 17, 20]: age at diagnosis (< 50 year, ≥ 50 year); nodal status (1–3, ≥ 4); grade [G1 (well differentiated), G2 (moderately differentiated), G3 (poorly differentiated), and undefined (GX)]; tumor size [T1 (≤ 2 cm), T2 (2 cm < size ≤ 5 cm), T3 (> 5 cm)]; ER status by IHC [negative (Allred score < 3), positive (Allred score ≥ 3)]; PR status by IHC [negative (Allred score < 3), positive (Allred score ≥ 3)], HER2 status by CISH [negative (Her2 gene to chromosome 17 ratio < 2), positive (ratio ≥ 2)]; Ki-67 by IHC (< 14% with nuclear staining of Ki67, ≥ 14%); and intrinsic subtypes (Luminal A, Luminal B, HER2-enriched, Basal-like) according to RT-qPCR algorithm for subtype prediction. Full details of these classifications can be found elsewhere [16, 17].

### Statistical analysis

We used a prospective-retrospective design (retrospective analysis of a randomized prospective trial) with pre-specified study objectives and pre-specified variable definitions. Our primary objective was to determine whether PAM50 breast tumor dimensions were associated with DFS and/or predictive of paclitaxel benefit. With a substantially greater number of events, the study has more power for DFS: 187 OS events compared to 283 events for DFS. For completeness, corresponding results for overall survival (OS) can also be found in Supplemental Material.

Univariable and multivariable Cox proportional hazards (PH) models were used to assess evidence for association with survival and interaction between treatment and quantitative dimensions. C-statistics are presented to illustrate discriminatory accuracy. Multivariable Cox PH using a stepwise procedure was used to determine an optimal model, with a Bayesian information criterion (BIC) penalty to balance the number of parameters in the model with the number of events [21]. To illustrate survival over time, non-parametric Kaplan–Meier estimates and survival curves were used, with a log-rank test to statistically compare survival between groups. All analyses were performed using R version 3.4.4, implementing version 2.42.3 of the ‘survival’ package. Results are presented consistent with the REMARK criteria for tumor marker prognostic studies [22].

## Results

### Patient characteristics

Gene expression data for the 50 classifier genes in the PAM50 assay were available for 820 of 1246 patients from the GEICAM/9906 trial. The clinical–pathological characteristics for this sub-study remains largely representative of the overall study population [16, 17]. Of the 820 patients with PAM50 data, one statistical outlier was removed. Descriptive statistics of the clinical–pathological variables and PAM50 categorical intrinsic subtypes using pre-specific categorization, by trial arm, are shown in Table 1. Tumor dimension summary statistics by arm are shown in Table S2. All patient characteristics were generally well balanced across the two arms of the trial.

**Table 1:** Patient characteristics

Variable	Arm				Total sub-study	
	FEC		FEC-P			
	*N* = 417	50.9 (%)	*N* = 402	49.1 (%)	*N* = 819	100 (%)
Age (years)						
< 50	197	47.2	197	49.0	394	48.1
≥ 50	220	52.8	205	51.0	425	51.9
Nodal Status						
1–3	257	61.6	249	61.9	506	61.8
4+	160	38.4	153	38.1	313	38.2
Histologic grade						
G1	54	12.9	54	13.4	108	13.2
G2	175	42.0	162	40.3	337	41.1
G3	160	38.4	155	38.6	315	38.5
GX	28	6.7	31	7.7	59	7.2
Primary tumor size						
T1	158	37.9	184	45.8	342	41.8
T2	236	56.6	195	48.5	431	52.6
T3	23	5.5	23	5.7	46	5.6
Estrogen receptor (IHC)						
Negative	95	22.8	77	19.2	172	21.0
Positive	320	76.7	323	80.3	643	78.5
Progesterone receptor (IHC)						
Negative	143	34.3	103	25.6	246	30.0
Positive	271	65.0	297	73.9	568	69.4
Her2 status (CISH)						
Negative	368	88.2	328	81.6	696	85.0
Positive	45	10.8	71	17.7	116	14.2
Ki-67 (IHC)						
Negative	278	66.7	279	69.4	557	68.0
Positive	132	31.7	110	27.4	242	29.5
PAM50 intrinsic subtypes						
Luminal A	131	31.4	149	37.1	280	34.2
Luminal B	144	34.5	117	29.1	261	31.9
HER2-enriched	84	20.1	91	22.6	175	21.4
Basal-like	45	10.8	26	6.5	71	8.7
Normal-like	13	3.1	19	4.7	32	3.9

Quantitative tumor dimensions for the GEICAM/9906 samples replicated the patterns observed in the LACE/PW discovery set [1]. Specifically, dimensions PC1, PC2, and PC4 together quantitatively echoed the standard categorical intrinsic subtype groupings in 3-dimensional space and illustrated the lack of mutual exclusivity of these categories (Fig. S1). As seen previously [1], the dimensions PC3 and PC5 represented tumor expression diversity beyond intrinsic subtypes and differ little by subtype. Figure S1 shows stacked histograms for PC3 and PC5 by subtype for the GEICAM/9906 samples and the discovery LACE/PW samples.

### Disease-free survival outcomes

The median follow-up of our sub-study of the GEICAM/9906 trial was 8.7 years. Consistent with the full trial data findings [13], DFS was significantly improved in the FEC-P arm in a univariable analysis [Cox PH hazard ratio [HR] for DFS of 0.75, 95% confidence interval (CI) 0.59–0.95, *p* = 0.016, Table 2]. Five-year DFS rates were 79% and 71% for FEC-P and FEC, respectively. In the FEC arm, 80% of women survived to 3.08 years, compared to 4.89 years in the FEC-P arm (log-rank *p* = 0.015) (Fig. S2).

**Table 2:** Univariate Cox proportional hazards for disease-free survival

Variable	Type	Reference	Tested	*n*	HR	95% CI		*p*	Wald *p*
Clinical trial									
Treatment arm	Categorical	FEC	FEC-P	819	0.75	0.59	0.95	0.016	0.016
Intrinsic dimensions									
PC1	Quantitative	na		819	1.32	1.18	1.46	6.7E × 10^−7^	6.7 × 10^−7^
PC2	Quantitative	na		819	0.93	0.82	1.04	0.21	0.21
PC3	Quantitative	na		819	0.93	0.83	1.05	0.24	0.24
PC4	Quantitative	na		819	1.08	0.96	1.22	0.21	0.21
PC5	Quantitative	na		819	0.88	0.78	0.99	0.036	0.036
Clinical–pathological characteristics									
Age at d*x*	Categorical	Onset ≥ 50	Onset < 50	819	1.12	0.89	1.42	0.32	0.32
Nodal status	Categorical	1–3	4 +	819	1.72	1.36	2.17	5.2 × 10^−6^	5.2 × 10^−6^
Histologic grade	Categorical	G1	G2	819	1.97	1.25	3.10	0.0036	0.0011
			G3		2.39	1.52	3.76	1.7 × 10^−4^	
			GX		1.48	0.79	2.79	0.22	
Tumor size	Categorical	T1	T2	819	1.67	1.29	2.15	9.3 × 10^−5^	7.2 × 10^−6^
			T3		2.59	1.65	4.07	3.5 × 10^−5^	
ER status	Categorical	Positive	Negative	815	1.33	1.01	1.74	0.04	0.04
PR status	Categorical	Positive	Negative	814	1.48	1.16	1.89	0.0014	0.0014
Her2 status	Categorical	Negative	Positive	812	1.57	1.16	2.12	0.003	0.003
Ki-67 status	Categorical	< 14%	≥ 14%	799	1.43	1.12	1.82	0.0044	0.0044
Intrinsic subtypes									
Subtype	Categorical	Luminal A	Luminal B	819	1.82	1.34	2.47	1.4 × 10^−4^	1.5 × 10^−5^
			Her2-enriched		2.20	1.58	3.05	2.5 × 10^−6^	
			Basal-like		2.08	1.35	3.22	9.4 × 10^−4^	
			Normal-like		1.02	0.49	2.13	0.95	

Hazard ratios for categorical variables are comparisons to the reference category, as noted. Hazard ratios for quantitative traits are per standard deviation (SD) unit for the trait

#### Prognosis (trial arms combined)

**Fig. 1:**
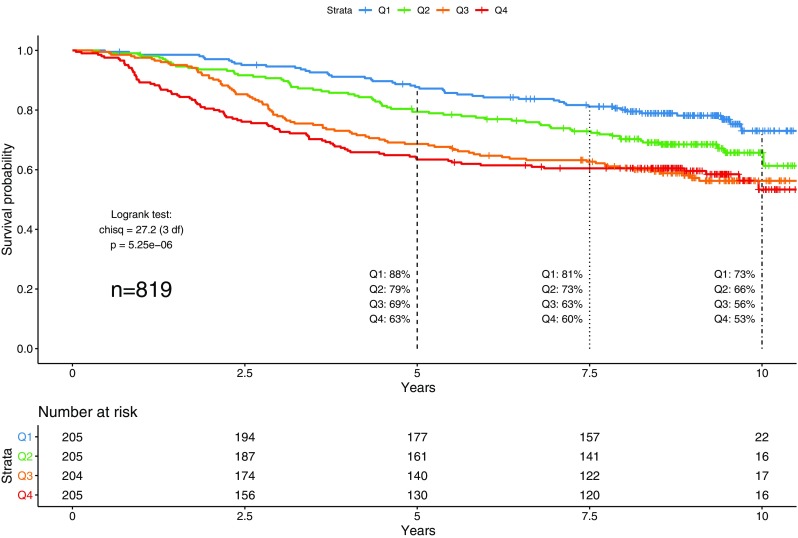
Disease-free survival by PC1 quartile. The blue Kaplan–Meier curve indicates DFS for women with tumors in the lowest quartile for PC1 (“Q1”); similarly, the green, orange, and red curves correspond to the second, third, and fourth quartiles (“Q2,” “Q3,” and “Q4”), respectively. Number of Patients at Risk for each group are shown below the plot

Univariable Cox PH for DFS for the five quantitative dimensions are shown in Table 2. Univariable analyses for clinical–pathological variables and categorical intrinsic subtypes were also performed. Dimension PC1 was associated with DFS (HR 1.32 95% CI 1.18–1.46, *p* = 6.7 $\times$ 10^−7^), indicating a highly significant 32% increased risk of recurrence per standard deviation increase in PC1. Notably, PC1 was the most significant predictor of DFS survival in univariable models, and remained significant after correction for all other clinical–pathological characteristics in multivariable models (Table 3). Figure 1 illustrates DFS by PC1 using a Kaplan–Meier plot for PC1-quartiles [Q1 (lowest quartile) to Q4 (highest quartile)]. Five-year DFS in the four quartiles were 88%, 79%, 69%, and 63% for quartiles Q1–Q4, respectively (log-rank *p* = 5.2 $\times$ 10^−6^). Dimension PC5 was also associated with DFS (HR 0.88 95% CI 0.78–0.99, *p* = 0.036), decreasing risk with increasing value, and remained similarly significant when corrected for all other clinical–pathological variables (Table S3).

**Table 3:** Multivariable Cox proportional hazards for disease-free survival

Model	Variables	Type	Reference	Tested	*n*	HR	95% CI		p	Wald *p*
PC1 + age at d*x*	PC1	Quantitative	na		819	1.31	1.18	1.46	8.3 × 10^−7^	3.0 × 10^−6^
	Age at d*x*	Categorical	Onset ≥ 50	Onset < 50		1.10	0.87	1.39	0.43	
PC1 + nodal status	PC1	Quantitative	na		819	1.31	1.17	1.47	1.5 × 10^−6^	5.4 × 10^−10^
	Nodal status	Categorical	1–3	4+		1.68	1.33	2.13	1.2 × 10^−5^	
PC1 + grade	PC1	Quantitative	na		819	1.26	1.11	1.43	0.0003	6.2 × 10^−6^
	Histologic grade	Categorical	G1	G2		1.79	1.13	2.83	0.013	
				G3		1.74	1.07	2.83	0.027	
				GX		1.36	0.72	2.57	0.34	
PC1 + tumor size	PC1	Quantitative	na		819	1.28	1.15	1.43	1.1 × 10^−5^	2.2 × 10^−9^
	Tumor size	Categorical	T1	T2		1.55	1.20	2.00	9.2 × 10^−4^	
				T3		2.45	1.56	3.84	1.0 × 10^−4^	
PC1 + ER status	PC1	Quantitative	ER		815	1.37	1.20	1.57	4.8 × 10^−6^	2.4 × 10^−6^
	ER status	Categorical	Positive	Negative		0.85	0.60	1.19	0.35	
PC1 + PR status	PC1	Quantitative	na		814	1.31	1.14	1.50	8.3 × 10^−5^	1.7 × 10^−6^
	PR status	Categorical	Positive	Negative		1.06	0.79	1.43	0.70	
PC1 + Her2-status	PC1	Quantitative	na		812	1.30	1.16	1.45	4.7 × 10^−6^	6.3 × 10^−7^
	Her2 status	Categorical	Negative	Positive		1.34	0.99	1.82	0.062	
PC1 + Ki-67 status	PC1	Quantitative	na		799	1.28	1.13	1.45	8.1 × 10^−5^	5.8 × 10^−6^
	Ki-67 status	Categorical	< 14%	≥ 14%		1.11	0.84	1.47	0.45	
PC1, adjusted for all clinical–pathological characteristics	PC1	Quantitative	na			1.27	1.07	1.50	0.0070	8.8 × 10^−8^
	Age at d*x*	Categorical	Onset ≥ 50	Onset < 50		1.05	0.83	1.34	0.6838	
	Nodal status	Categorical	1–3	4 +		1.44	1.13	1.84	0.0035	
	Histologic grade	Categorical	G1	G2		1.55	0.97	2.48	0.0649	
				G3		1.40	0.85	2.30	0.1859	
				GX		1.07	0.56	2.03	0.8451	
	Tumor size	Categorical	T1	T2		1.36	1.04	1.77	0.0232	
				T3		2.10	1.30	3.41	0.0026	
	ER status	Categorical	Positive	Negative		0.81	0.56	1.18	0.2773	
	PR status	Categorical	Positive	Negative		1.14	0.82	1.59	0.4468	
	Her2 status	Categorical	Negative	Positive		1.21	0.88	1.67	0.2325	
	Ki-67 status	Categorical	< 14%	≥ 14%		1.02	0.77	1.35	0.8807	

Hazard ratios for categorical variables are comparisons to the reference category, as noted. Hazard ratios for quantitative traits are per standard deviation (SD) unit for the trait

Multivariable analysis with selection from all variables (including categorical intrinsic subtypes) identified the optimal model for DFS to include PC1 (HR 1.32, *p* = 1.3 $\times$ 10^−6^); nodal status (HR 1.56, *p* = 2.7 $\times$ 10^−4^); PC5 (HR 0.83, *p* = 3.0 $\times$ 10^−3^); and tumor size (T2: HR 1.39, *p* = 0.013; T3: HR 2.16, *p* = 1.0 $\times$ 10^−3^), with a highly significant overall fit (*p* = 2.5 $\times$ 10^−12^, c-statistic = 0.65). The best-fitting model without considering dimensions, but still considering categorical subtypes, included nodal status, tumor size, and PR status (*p* = 1.0 $\times$ 10^−7^, c-statistic = 0.61). Categorical subtypes are not selected.

#### Treatment interactions

**Fig. 2:**
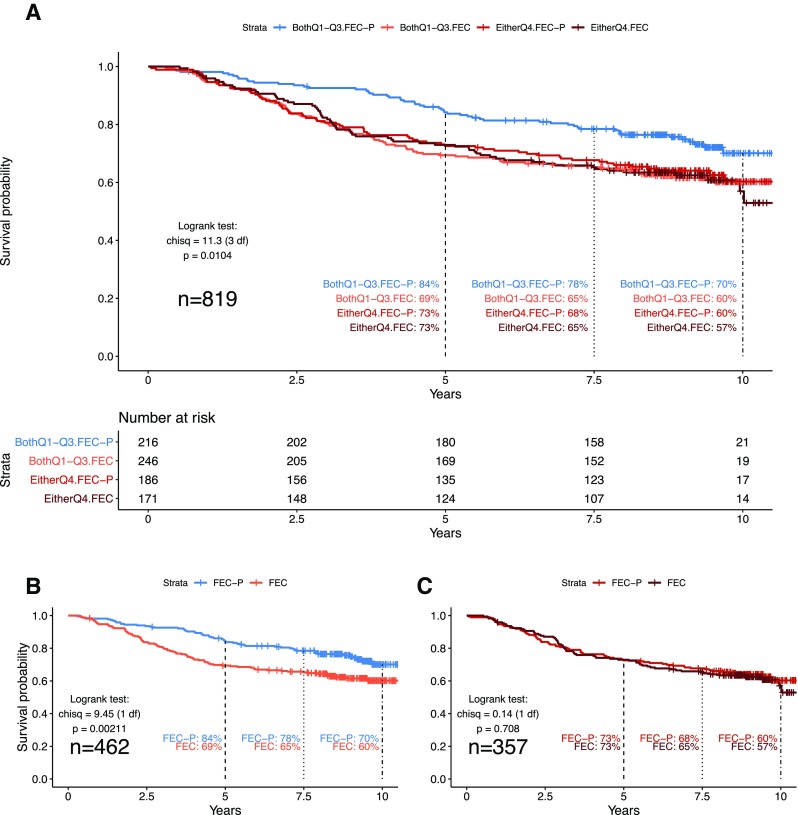
Disease-free survival by treatment arm and PC3/PC4 quartile. **a** Kaplan–Meier curves for the following four groups: low PC3 and PC4 and FEC plus paclitaxel treatment (“BothQ1–Q3.FEC-P”); low PC3 and PC4 and FEC treatment only (“BothQ1–Q3.FEC”); high in either PC3 or PC4 and FEC plus paclitaxel treatment (“EitherQ4.FEC-P”); high in either PC3 or PC4 and FEC treatment only (“EitherQ4.FEC”). Number of Patients at Risk for each group are shown below the plot. **b** Kaplan–Meier curves for FEC versus FEC plus paclitaxel for tumors that are low PC3 and PC4. **c** Kaplan–Meier curves for FEC versus FEC plus paclitaxel for tumors that are high in PC3 or PC4

Cox PH models including trial arm (FEC or FEC-P) were used to identify the potential for interactions between the five quantitative dimensions and treatment (Table 4). Parallel analyses for clinical–pathological variables and categorical intrinsic subtypes were also performed (Table S4). Dimensions PC3 and PC4 indicated interactions with treatment arm. No other variables had significant interaction effects. Further, the model including PC3, PC4, and treatment arm indicated the two dimension–treatment interactions were independent effects (PC3*treatment HR_int_ = 1.28 95% CI 1.02–1.62, *p* = 0.037; PC4*treatment HR_int_ = 1.28 95% CI 1.01–1.63, *p* = 0.045). Both interaction hazard ratios are > 1.0, representing increasing hazards for the addition of paclitaxel (FEC-P) with increasing values of PC3 or PC4. These interactions provide the potential to nullify the positive effect of treatment for high PC3 or PC4 values, and conversely, to amplify the positive effect of treatment for low PC3 or PC4 values. Figure 2a illustrates differences in DFS using a Kaplan–Meier plot with four groups: treatment arm (FEC; FEC-P) by PC3 and PC4 values (highest quartile [Q4] of PC3 or PC4; lower quartiles [Q1–Q3] for both PC3 and PC4). For tumors low for both PC3 and PC4, the 5-year DFS was 84% and 69% for FEC-P and FEC arms, respectively, and for tumors high for either PC3 or PC4, 5-year DFS was 73% and 73% for FEC-P and FEC (log-rank *p* = 0.010). To further illustrate these interactions, Fig. 2b illustrates the amplified and significant improvement in DFS in the FEC-P arm for women whose tumors were low for PC3 and PC4 (log-rank *p* = 0.0021). In the FEC arm, 80% of women survived to 3.03 years, compared to 7.06 years in the FEC-P arm. Consistent with this, a Cox PH survival analysis for women with low PC3/PC4 tumors showed HR 0.60 for FEC-P (*p* = 0.0024), which remained stable and significant after adjustment for other significant clinical–pathological variables (FEC-P HR 0.63, *p* = 0.0074; nodal status HR 1.75, *p* = 6.9 $\times$ 10^−4^; PR-status HR 1.60, *p* = 0.0061). In contrast, Fig. 2c shows the null result for DFS and addition of paclitaxel for women who had tumors with high values for either PC3 or PC4 (log-rank *p* = 0.71). For high PC3/4, 80% of women survived to 3.16 years in the FEC arm, compared to 3.10 years in the FEC-P arm. The companion Cox PH survival analysis for women with high PC3/PC4 tumors showed that the lack of effect of treatment continued after adjustment for other significant clinical–pathological variables (FEC-P HR 0.91, *p* = 0.57; tumor size T2 HR 1.56, *p* = 0.018; tumor size T3 HR 2.96, *p* = 2.9 $\times$ 10^−4^). In both high and low PC3/PC4 groups, the women in the FEC arm have comparable survival, but only those women with low PC3 and PC4 tumors show improved survival with the addition of paclitaxel. Of the 819 women in the sub-study, 462 (56.4%) have low PC3 and PC4 tumors, and 357 women (43.6%) have tumors high for either PC3 or PC4. The potential impact of lack of effect (43.6%) is large.

**Table 4:** Multivariable Cox proportional hazards for interaction of intrinsic dimensions with treatment

Model	Variables	Type	Reference	Tested	*n*	HR	95% CI		*p*	Interaction *p*
Intrinsic dimensions										
Treatment ARM + PC1	Treatment arm*PC1	Interaction			819	1.09	0.88	1.36		0.43
	Treatment arm	Categorical	FEC	FEC-P		0.78	0.61	0.99	0.038	
	PC1	Quantitative	na			1.25	1.09	1.45	0.0021	
Treatment ARM + PC2	Treatment arm*PC2	Interaction			819	0.88	0.70	1.12		0.31
	Treatment arm	Categorical	FEC	FEC-P		0.75	0.59	0.94	0.015	
	PC2	Quantitative	na			0.99	0.84	1.16	0.86	
Treatment ARM + PC3	Treatment arm*PC3	Interaction			819	1.26	1.00	1.60		0.052
	Treatment arm	Categorical	FEC	FEC-P		0.75	0.59	0.95	0.018	
	PC3	Quantitative	na			0.85	0.73	0.98	0.028	
Treatment ARM + PC4	Treatment arm*PC4	Interaction			819	1.28	1.00	1.62	0.048	0.048
	treatment arm	Categorical	FEC	FEC-P		0.73	0.58	0.93	0.011	
	PC4	Quantitative	na			0.96	0.81	1.14	0.68	
Treatment ARM + PC5	Treatment arm*PC5	PC5			819	1.13	0.88	1.44		0.34
	Treatment arm	Categorical	FEC	FEC-P		0.75	0.60	0.95	0.019	
	PC5	Quantitative	na			0.84	0.72	0.98	0.023	
Treatment ARM + PC3 + PC4	Treatment arm*PC3	Interaction			819	1.28	1.02	1.62		0.037
	Treatment arm*PC4	Interaction				1.28	1.01	1.63		0.045
	Treatment arm	Categorical	FEC	FEC-P		0.74	0.58	0.94	0.012	
	PC3	Quantitative	na			0.85	0.73	0.98	0.029	
	PC4	Quantitative	na			0.97	0.82	1.15	0.738	

Hazard ratios for categorical variables are comparisons to the reference category, as noted. Hazard ratios for quantitative traits are per standard deviation (SD) unit for the trait

Clinical–pathological characteristics of high PC3 tumors (i.e., PC3 Q4), compared to the overall dataset (greater than 5% absolute difference), show these are more likely to be lower grade and smaller tumors, ER+, PR+, Her2−, low Ki67, and Luminal A subtype, and in women with d*x* < 50 years (Table S5). Kaplan–Meier curves of PC3 by treatment show that women with high PC3 tumors are good responders to the standard FEC treatment (5-year DFS of 78%), and gain no further improvement with the addition of paclitaxel (5-year DFS of 77%) (Fig. S3). Relative to the high PC3 tumors, low PC3 tumors (i.e., PC3 Q1–Q3) are therefore more likely to be higher grade and larger size, and be ER−, PR−, Her2+, Luminal B or Basal-like subtypes, and onset $\ge$ 50 year (Table S5). Women with these low PC3 tumors benefit from paclitaxel (5-year DFS 69% FEC, 79% FEC-P) (Fig. S3).

Clinical–pathological characteristics of high PC4 tumors compared to the overall dataset show these are more likely to be higher grade tumors, more nodes involved, and be ER−, PR−, Her2+, and more likely to be the HER2-enriched subtype (Table S5). Kaplan–Meier curves for PC4 and treatment show that women with high PC4 tumors are poorer responders to standard FEC treatment (5-year DFS of 70%), and that this poor response persists, even with FEC-P (5-year DFS of 67%) (Fig. S4). Relative to the high PC4 tumors, low PC4 (i.e., PC4 Q1–Q3) tumors are therefore more likely to have fewer nodes, lower grade, and be ER+, PR+, Her2−, and Luminal A subtype (Table S5). Women with low PC4 tumors benefit from paclitaxel (5-year DFS 71% FEC, 83% FEC-P) (Fig. S4).

## Discussion

The focus of this study was proof-of-concept of the utility of quantitative tumor dimensions in a clinical trial setting, with application to the GEICAM/9906 breast cancer cohort and investigation of association with prognosis or response to paclitaxel. Previous work with this cohort investigated PAM50 categorical intrinsic subtypes and an 11-gene quantitative proliferation score [16]. That previous analysis showed that nodal status, tumor size, and the 11-gene proliferation score (*p* = 0.013), but not categorical subtypes, were significant independent predictors of overall survival [16]. The proliferation score also indicated interaction with treatment response, with tumors low on the proliferation score gaining benefit from the addition of weekly paclitaxel [16].

Here, we investigated agnostically derived quantitative dimensions for association with survival and response. In multivariable analysis we found that nodal status, tumor size, PC1 (*p* = 1.3 $\times$ 10^−6^), and PC5 (*p* = 3.0 $\times$ 10^−3^) were significant independent predictors for DFS. Notably, PC1 was the most significant predictor, superior to established clinical–pathological characteristics and categorial intrinsic subtypes. Importantly, PC1 and PC5 are independent prognostic indicators, remaining significant in multivariable models corrected for the established indicators. PC1 is a quantitative tumor trait that compiles many poor classical prognostic indicators, such as being ER/PR negative, and higher grade and proliferation (Fig. S5, Table S6). PC1 has a much weaker relationship with nodal status and tumor size, which likely explains why the best-fitting global hazard model includes both PC1 and these other predictors. We anticipate that PC1 is efficiently capturing information that is also reflected in earlier gene expression prognostic indicators used for ER+ breast cancer [3, 7, 8]. PC5 is an intriguing novel finding. It is not correlated with clinical–pathological characteristics (Fig. S5), or intrinsic subtype [1] (Table S6). It therefore represents a new tumor trait that provides additional independent information regarding prognosis.

Both this current study and the previous study [16] of GEICAM/9906 find categorical intrinsic subtypes are not independently prognostic in multivariable analyses adjusting for standard clinical–pathological variables. Similarly, both studies find that quantitative traits derived from the PAM50 genes have potential clinical utility as additional independent predictors. In Martin *and colleagues* [16], the expert-curated 11-gene proliferation score provides a simple average of the expression for 11 genes selected for known functional involvement in proliferation. An advantage of an expert-curated trait is its interpretation, and a disadvantage its restriction to current knowledge. Conversely, the tumor dimensions explored here are agnostic to gene function. Principal components analysis derives the traits, which are weighted averages across all 50 genes (weights can be positive or negative). Advantages include the increased number of quantitative traits to explore (prior to understanding their molecular interpretation) and that these are independent lending themselves to statistical modeling. Here, the advantages are underscored by the fact that PC1 was found to be the most significant single best predictor for prognosis in multivariable analyses (capturing more of the association than previously found using the expert-curated 11-gene proliferation score), and that a second dimension, PC5, may also be of independent prognostic importance.

We also explored the utility of quantitative tumor dimensions for association with response to treatment. Studies of interactions are challenging due to the increased sample size required. Clinical trials are powered to meet a therapeutic endpoint and not a diagnostic biomarker endpoint. Nonetheless, in a formal multivariable statistical test, we found nominal significance, and independent interactions between treatment arm and both PC3 (*p* = 0.037) and PC4 (*p* = 0.045), indicating the potential for countering the FEC-P effect in high PC3 and high PC4 tumors. The Kaplan–Meier illustration of this was dramatic (Fig. 2a-c), suggesting that only women with tumors low for PC3 and PC4 (specifically, Q1–Q3) responded to the addition of paclitaxel.

The mechanisms for the two independent interaction effects identified for PC3 and PC4 are likely to be very different. The pattern of response found for PC3 is consistent with potential over-treatment of good prognosis disease, while the pattern for PC4 is consistent with the identification of a group of generally resistant tumors. Novel quantitative dimensions can differentiate these two independent effects, thus providing new avenues to pursue regarding precision medicine and paclitaxel use. In particular, it highlights high PC3 tumors as a subset of patients who could avoid the potential toxicity of an unnecessary additional chemotherapy, and equally importantly highlights high PC4 tumors as a subset of patients where it will be critical to find alternate treatment strategies. For example, women with high PC4 tumors could forego a taxane for biologic therapy such as targeted Her2 therapy (e.g., trastuzumab or lapatinib). Genes *ERBB2 and GRB7* (genes known to be DNA amplified together on chromosome 17q12) are among those that are prominent in the PC4 gene coefficients (Table S1).

Of interest are the seemingly opposing conclusions of the interaction effects if interpreted using only standard clinical–pathological characteristics. As described in Results and shown in Table S5, high PC3 tumors are lower grade, smaller tumors, and more likely to be ER+, PR+, Her2−, and low Ki67. Figure S3 shows that women with these tumors are already good responders to the standard of care FEC and do not further respond to the addition of paclitaxel (5-year DFS 78% FEC, 77% FEC-P). In *comparison* to the high PC3 tumors, low PC3 tumors are higher grade, larger size, and be ER−, PR−, and Her2+, and do respond to the addition of paclitaxel (5-year DFS 69% FEC, 79% FEC-P) (Fig. S3). Seemingly in contradiction with these PC3 findings are that high PC4 tumors have more nodal involvement, are higher grade tumors, and more likely to be ER−, PR−, and Her2+. Women with these types of tumors are poor responders to standard FEC treatment with no benefit to the addition of paclitaxel (5-year DFS 70% FEC, 67% FEC-P) (Fig. S4). In *comparison to* the high PC4 tumors, low PC4 tumors have fewer nodes, lower grade, and are more likely to be ER+, PR+, Her2−, and respond to the addition of paclitaxel (5-year DFS 71% FEC, 83% FEC-P) (Fig. S4). If we compare these findings to those presented in the literature, the low PC3 responders are consistent with previous results for positive paclitaxel response in Her2+/ER− tumors [10], yet the high PC4 non-responders (also more likely to be Her2+/ER−) are consistent with non-interaction of HER2 status as found in another study [13]. Similarly, the low PC4 responders appear consistent with previous results for positive paclitaxel response in low proliferative tumors [16] (ER+, PR+, Her2−, low grade, small tumors), yet the high PC3 non-responders are also more likely to be these low-risk tumors. While within each context there are comparative differences in clinical–pathological characteristics that can be made—these are not defining features. This illustrates a complexity that is captured by dimensions. The contrary interpretations are simply that clinical–pathological variables are poor proxies for dimension values. Hence, quantitative dimensions provide the opportunity to differentiate tumors beyond standard criteria and the potential to resolve apparently conflicting prior results.

There are limitations to this study. Although the PC dimensions applied in this study were pre-defined on an independent cohort of breast cancer patients (training set), the prognostic and predictive findings using these dimensions should be confirmed. In addition, we have explored and found utility of tumor dimensions in a single study only, and it is possible that this utility is limited to the specific characteristics within the GEICAM/9906 trial. However, given our successful use of dimensions in another domain (germline gene mapping), we are confident that the approach will garner power otherwise being ignored. Many studies that have already performed the PAM50 assay can immediately re-analyze with dimensions (equations in Table S1 and Madsen et al. [1]).

In conclusion, we have presented the first study to consider utility of quantitative breast tumor dimensions in a clinical trial setting for association with prognosis and predictive value for response to treatment. Quantitative traits avoid the need to sub-divide the sample, thus maintaining sample size and, in general, more statistical power can be gained when quantitative traits are used in place of dichotomized variables. The five dimensions derived from the PAM50 gene set are quantitative and independent, providing a flexible framework for modeling tumor diversity in any study design. We previously showed that certain dimensions (and not intrinsic subtype) were powerful for gene mapping [1], suggesting they represent biological-relevant tumor characteristics. Here, we have illustrated that they have potential to provide prognostic and predictive information, including capturing aspects of the tumor that are not evident from classical clinical–pathological or categorical PAM50 subtyping. They also may provide potential to resolve conflicting findings previously based on clinical–pathological groupings. While our specific results require replication in independent similarly designed clinical trials, we believe there is clear potential for utility of these quantitative dimensions as novel, tumor traits that should be considered in future clinical trials in general.

## Electronic supplementary material

Below is the link to the electronic supplementary material.

Supplementary material 1 (PDF 2707 KB)

Supplementary material 2 (DOCX 13 KB)

## Abbreviations

GEICAM: Spanish Breast Cancer Group (Grupo Español para la Investigación del Cáncer de Mama)
FEC: Fluorouracil, epirubicin, and cyclophosphamide
FEC-P: FEC followed by weekly paclitaxel
FFPE: Formalin-fixed, paraffin-embedded
GEP: Gene expression profiling
ER: Estrogen receptor
PR: Progesterone receptor
HER2: Human epidermal receptor 2
IHC: Immunohistochemistry
CISH: Chromogenic in situ hybridization
DFS: Disease-free survival
PH: Proportional hazards
BIC: Bayesian information criterion
