# Supplementary material for: Re-interpretation of PAM50 gene expression as quantitative tumor dimensions shows utility for clinical trials: application to prognosis and response to paclitaxel in breast cancer
Source: Breast Cancer Res Treat. 2019 Jan 23;175(1):129–39. doi: 10.1007/s10549-018-05097-5 (PMC6491406; doi:10.1007/s10549-018-05097-5)
Supplement: Supplementary file 1 — Supplementary material 1 (PDF 2707 KB) [file 10549_2018_5097_MOESM1_ESM.pdf]

FIGURE S1

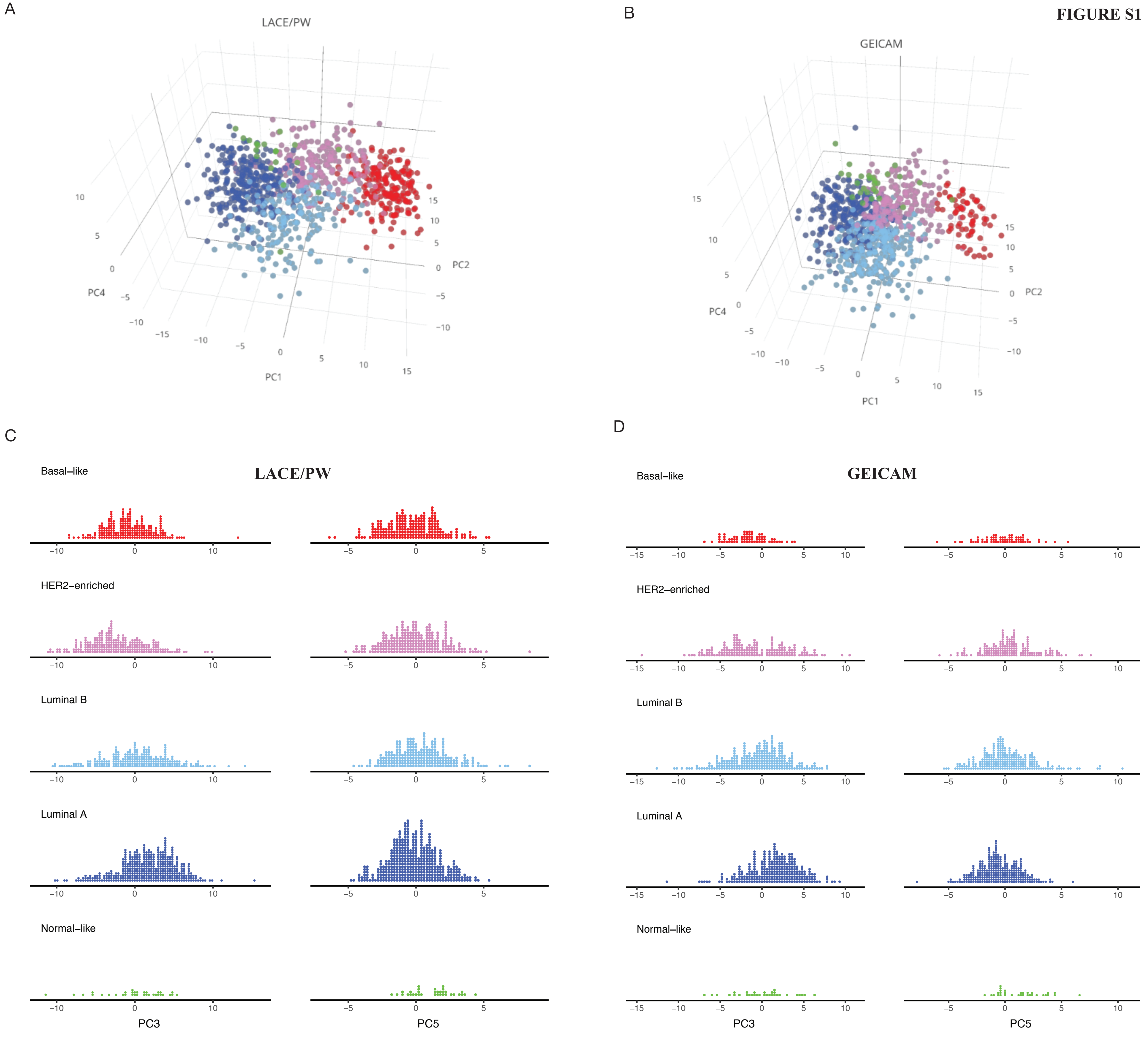
A
LACE/PW
PC1
PC2
PC4
B
GEICAM
PC1
PC2
PC4
C
LACE/PW
Basal–like
HER2–enriched
Luminal B
Luminal A
Normal–like
PC3
PC5
D
GEICAM
Basal–like
HER2–enriched
Luminal B
Luminal A
Normal–like
PC3
PC5

FIGURE S2

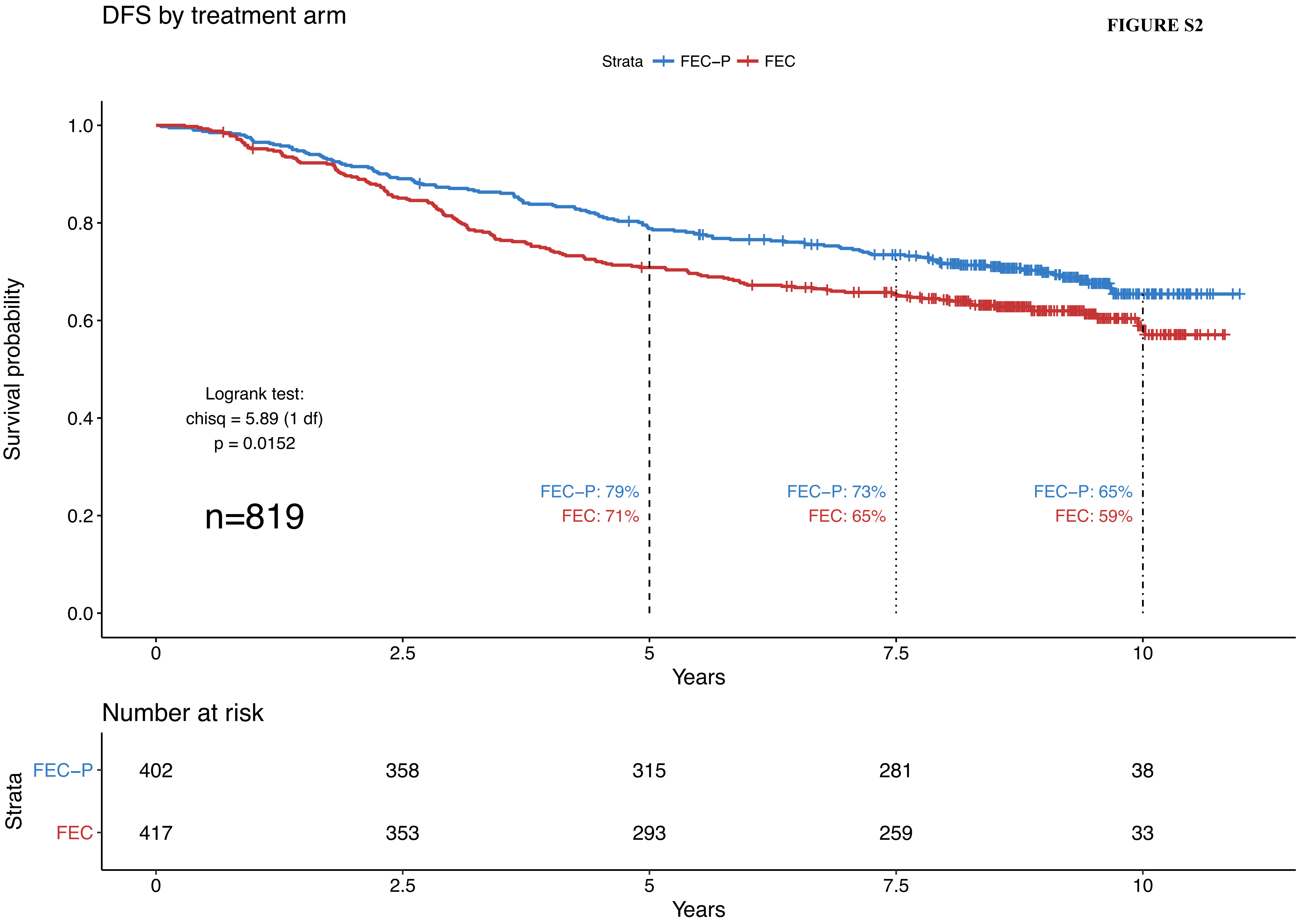
DFS by treatment arm
Strata FEC–P FEC
Survival probability
1.0
0.8
0.6
0.4
0.2
0.0
Logrank test:
chisq = 5.89 (1 df)
p = 0.0152
n=819
FEC–P: 79%
FEC: 71%
FEC–P: 73%
FEC: 65%
FEC–P: 65%
FEC: 59%
0
2.5
5
7.5
10
Years
Number at risk
Strata
FEC–P 402 358 315 281 38
FEC 417 353 293 259 33
0
2.5
5
7.5
10
Years

FIGURE S3

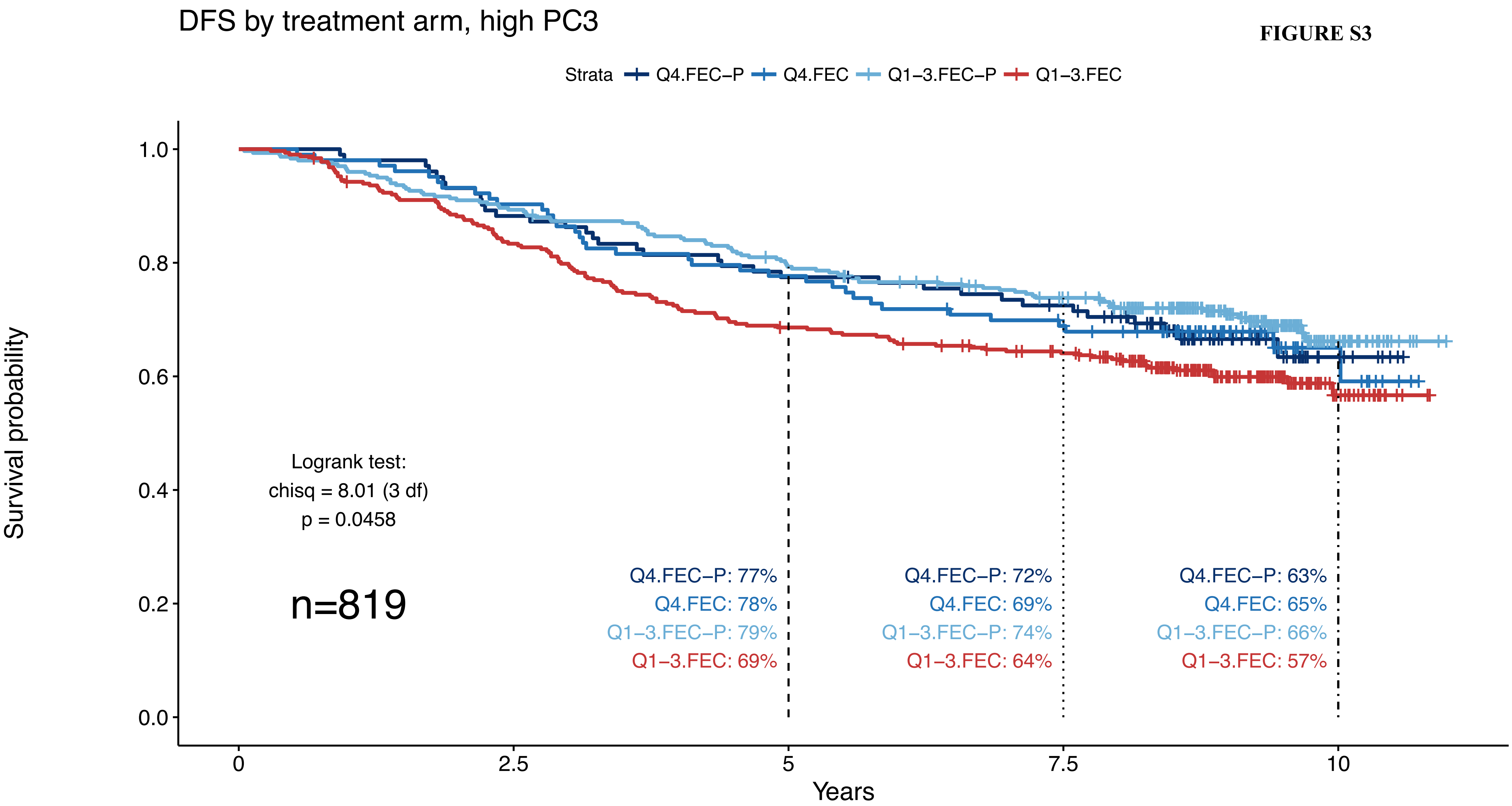

Strata	0	2.5	5	7.5	10
Q4.FEC−P	102	90	79	72	8
Q4.FEC	103	93	80	69	11
Q1−3.FEC−P	300	268	236	209	30
Q1−3.FEC	314	260	213	190	22

FIGURE S4

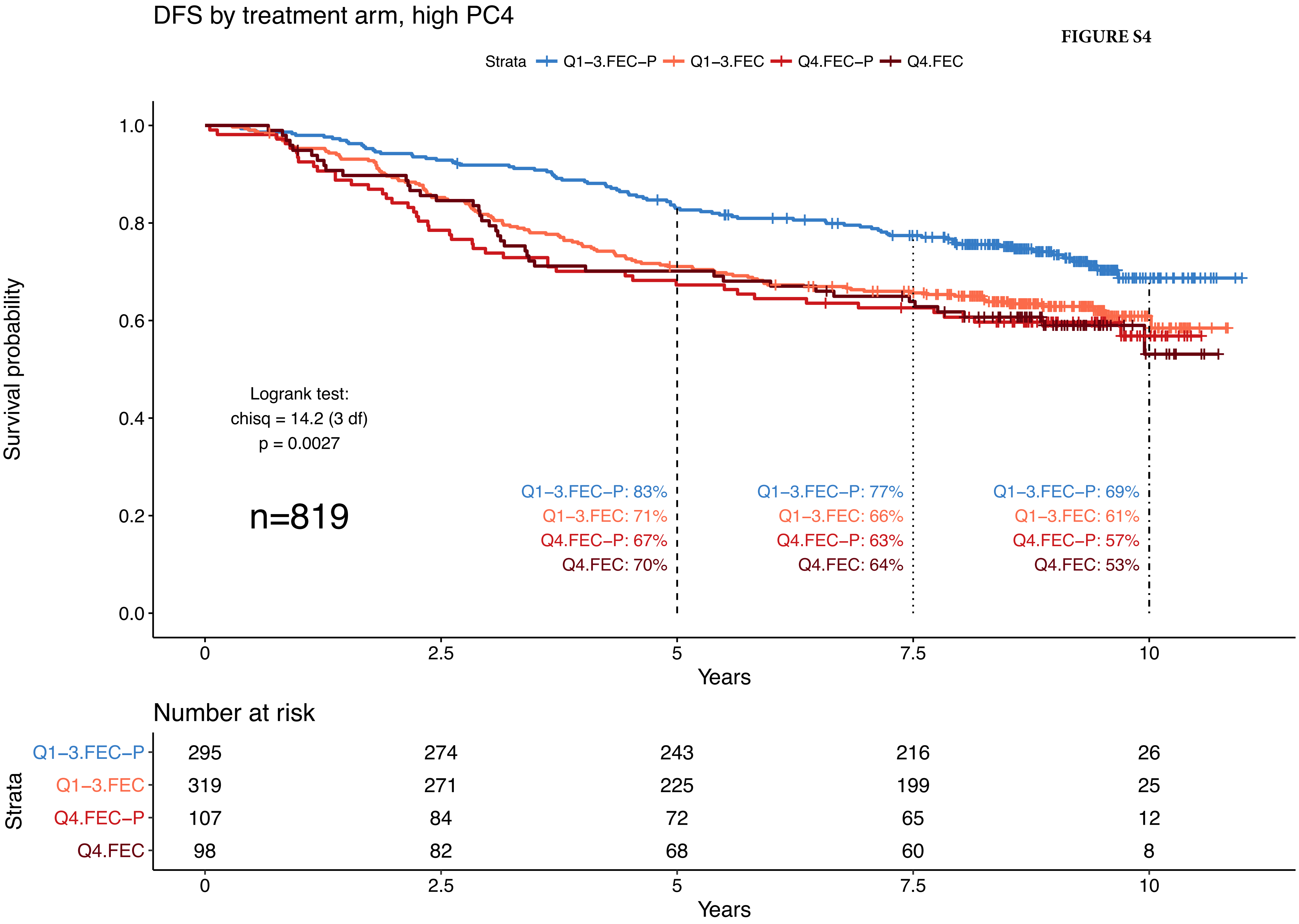
DFS by treatment arm, high PC4
Strata
Q1–3.FEC–P
Q1–3.FEC
Q4.FEC–P
Q4.FEC
Survival probability
1.0
0.8
0.6
0.4
0.2
0.0
Logrank test:
chisq = 14.2 (3 df)
p = 0.0027
n=819
Q1–3.FEC–P: 83%
Q1–3.FEC: 71%
Q4.FEC–P: 67%
Q4.FEC: 70%
Q1–3.FEC–P: 77%
Q1–3.FEC: 66%
Q4.FEC–P: 63%
Q4.FEC: 64%
Q1–3.FEC–P: 69%
Q1–3.FEC: 61%
Q4.FEC–P: 57%
Q4.FEC: 53%
0
2.5
5
7.5
10
Years
Number at risk
Strata
Q1–3.FEC–P 295 274 243 216 26
Q1–3.FEC 319 271 225 199 25
Q4.FEC–P 107 84 72 65 12
Q4.FEC 98 82 68 60 8
0
2.5
5
7.5
10
Years

FIGURE S5

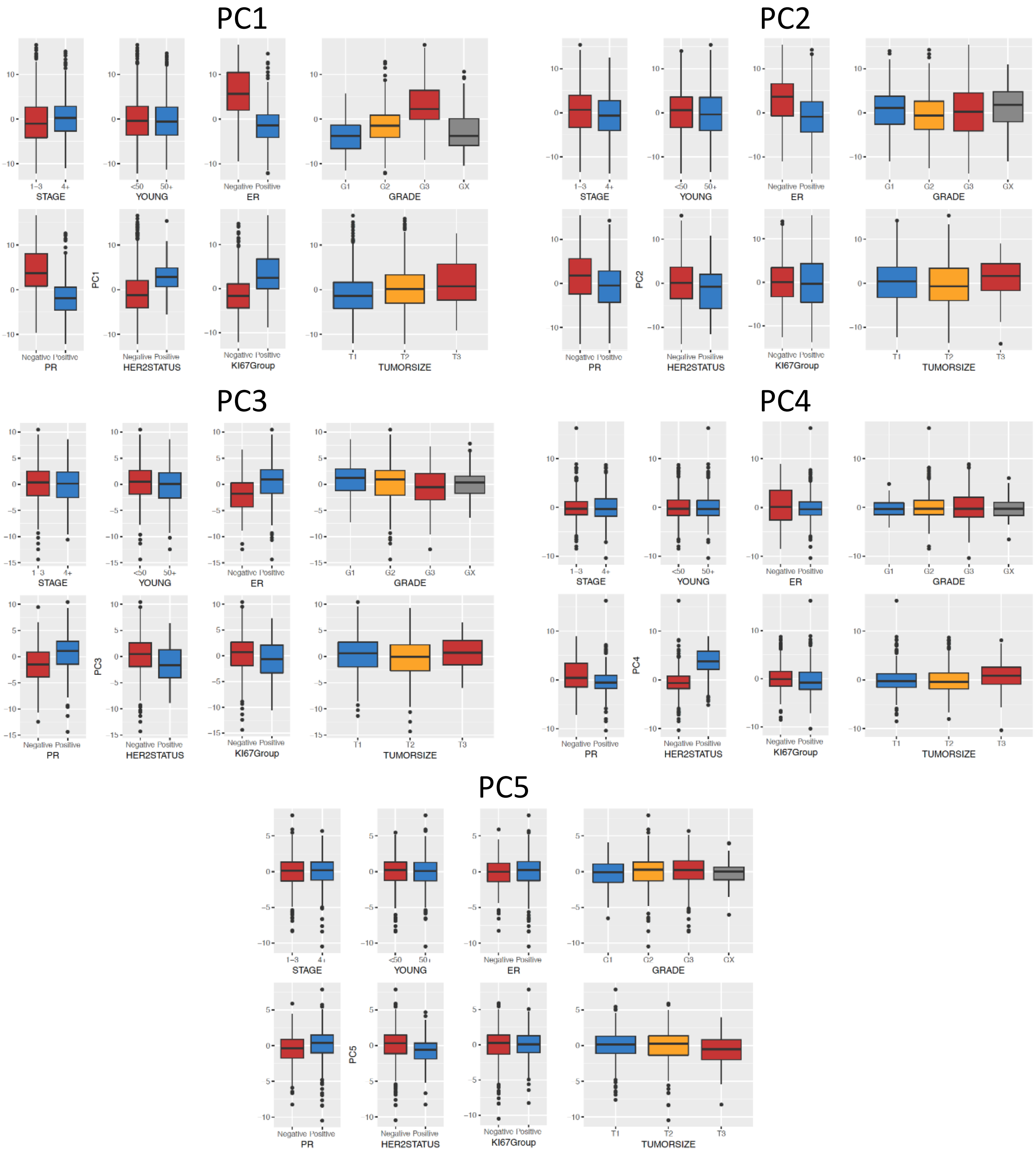
PC1
PC2
PC3
PC4
PC5
STAGE
YOUNG
ER
GRADE
PR
HER2STATUS
KI67Group
TUMORSIZE
1–3
4+
<50
50+
Negative Positive
G1
G2
G3
GX
T1
T2
T3

TABLE S1

	PC1	PC2	PC3	PC4	PC5
**Eigenvalues (variance)**	**40.4616**	**25.1278**	**13.509**	**7.0587**	**4.2068**
**%Cumul. Variance**	**0.3046**	**0.1892**	**0.1017**	**0.0531**	**0.0317**
**PGR**	-0.2811	-0.0954	0.335	-0.1923	0.2781
**ESR1**	-0.2514	-0.1858	0.2487	-0.1292	-0.0375
**NAT1**	-0.2342	-0.1189	0.2959	-0.0573	0.0444
**FOXA1**	-0.2186	-0.1888	0.1518	0.2437	-0.0639
**MLPH**	-0.1986	-0.1045	0.1694	0.1239	-0.2245
**MAPT**	-0.1752	-0.0928	0.1625	-0.0845	0.12
**MYBL2**	0.2323	-0.1256	0.0742	0.021	0.0861
**EXO1**	0.2025	-0.1507	0.1142	0.0118	-0.0671
**CDC20**	0.195	-0.0991	0.1046	-0.0051	-0.0022
**BIRC5**	0.1943	-0.1465	0.1389	-0.0002	-0.0306
**CEP55**	0.1816	-0.119	0.1292	-0.0005	0.0399
**ORC6L**	0.1807	-0.0569	0.0583	0.0231	0.0075
**RRM2**	0.1801	-0.1337	0.0978	0.014	0.0383
**MELK**	0.1754	-0.0643	0.0594	-0.0195	0.0805
**CCNE1**	0.1698	-0.0223	0.0008	0.022	0.0489
**KIF2C**	0.1682	-0.0867	0.0883	0.0214	-0.0355
**KNTC2**	0.1679	-0.0914	0.0903	-0.0444	0.069
**MKI67**	0.1589	-0.1065	0.1228	-0.0181	0.0556
**CDC6**	0.1579	-0.1392	0.0976	0.0774	-0.1266
**CDCA1**	0.1501	-0.1115	0.1506	-0.0239	0.0265
**KRT14**	-0.0235	0.393	0.3526	0.0603	-0.0022
**KRT5**	0.0528	0.3944	0.3062	0.1079	0.0518
**KRT17**	0.0888	0.3665	0.2469	0.1816	0.2391
**SFRP1**	0.0332	0.2693	0.0674	-0.1137	-0.26
**MIA**	0.0986	0.2368	0.1781	-0.1115	-0.1924
**SLC39A6**	-0.1161	-0.1015	0.2166	-0.0663	-0.133
**BCL2**	-0.0945	-0.0287	0.1505	-0.0457	-0.1773
**TMEM45B**	-0.1462	-0.042	-0.0488	0.5837	0.1404
**GRB7**	0.0522	-0.0671	0.0133	0.3506	-0.152
**ERBB2**	-0.0026	-0.0561	0.0216	0.3424	-0.0871
**FGFR4**	0.0301	-0.0458	0.0152	0.2116	-0.3159
**EGFR**	0.0342	0.1035	-0.0002	0.1595	-0.115
**GPR160**	-0.0852	-0.0756	0.0782	0.1564	-0.0929
**FOXC1**	0.0977	0.1095	0.0576	-0.0637	-0.224
**MMP11**	-0.0083	-0.0639	0.0137	0.1293	0.4308
**CDH3**	0.1146	0.1449	0.0801	0.1431	0.1431
**CXXC5**	-0.0536	-0.0893	0.1373	0.0164	-0.0827
**ANLN**	0.1341	-0.0563	0.0793	0.0291	0.07
**PHGDH**	0.1302	0.063	-0.0188	0.0031	0.0542
**MYC**	0.0401	0.0179	0.0254	-0.1238	-0.1296
**UBE2C**	0.1247	-0.1102	0.1111	-0.0192	-0.0298
**TYMS**	0.1238	-0.0691	0.0774	-0.0649	0.0554
**PTTG1**	0.1237	-0.0849	0.0731	-0.0183	-0.0199
**CCNB1**	0.1234	-0.0932	0.1055	0.0156	0.036
**MDM2**	-0.0249	-0.0192	0.0676	0.0141	-0.1066
**UBE2T**	0.0982	-0.0732	0.0963	0.0309	-0.0094
**CENPF**	0.071	-0.0268	0.091	0.0175	-0.0526
**BAG1**	-0.0241	-0.0154	0.0893	0.0118	-0.0329
**BLVRA**	-0.0189	-0.0435	0.0763	0.0704	-0.016
**ACTR3B**	-0.0132	-0.0063	0.0332	-0.0302	-0.0416

TABLE S2

INTRINSIC DIMENSIONS, DESCRIPTIVES												
Variable	Arm								Total sub-study (N=819, 100%)			
	FEC (N=417, 50.9%)				FEC-P (N=402, 49.1%)							
	Mean	s.d.	min.	max.	Mean	s.d.	min.	max.	Mean	s.d.	min.	max.
**PC1**	0.5	5.5	-12.0	16.6	-0.6	5.1	-12.1	15.2	0.0	5.4	-12.1	16.6
**PC2**	-0.1	5.3	-13.7	13.4	0.1	5.2	-13.5	15.4	0.0	5.3	-13.7	15.4
**PC3**	0.0	3.7	-14.4	10.4	0.0	3.5	-11.4	9.2	0.0	3.6	-14.4	10.4
**PC4**	-0.1	2.9	-10.4	16.3	0.1	2.8	-8.5	8.9	0.0	2.8	-10.4	16.3
**PC5**	0.0	2.3	-7.9	10.4	0.0	2.1	-5.5	8.4	0.0	2.2	-7.9	10.4

TABLE S3

MULTIVARIABLE COX PROPORTIONAL HAZARDS for DISEASE FREE SURVIVAL										
**Model**	**Variables**	**Type**	**Reference**	**Tested**	**n**	**HR**	**95% CI**		**p**	**Wald p**
**PC5 + Age at dx**										
	PC5	quantitative	na		819	0.88	0.78	0.99	0.037	0.070
	Age at dx	categorical	onset ≥50	onset<50		1.12	0.89	1.42	0.34	
**PC5 + Nodal status**										
	PC5	quantitative	na		819	0.88	0.78	0.99	0.030	2.9E-06
	Nodal status	categorical	1 - 3	4+		1.73	1.37	2.18	4.3E-06	
**PC5 + Grade**										
	PC5	quantitative	na		819	0.89	0.79	1.00	0.057	5.5E-04
	Histologic Grade	categorical	G1	G2		1.94	1.23	3.06	4.4E-03	
				G3		2.35	1.50	3.70	2.2E-04	
				GX		1.48	0.79	2.79	0.22	
**PC5 + Tumor Size**										
	PC5	quantitative	na		819	0.87	0.77	0.98	0.021	2.2E-06
	Tumor size	categorical	T1	T2		1.67	1.29	2.16	8.4E-05	
				T3		2.69	1.71	4.22	1.8E-05	
**PC5 + ER status**										
	PC5	quantitative	ER		815	0.88	0.78	0.99	0.031	0.013
	ER status	categorical	positive	negative		1.34	1.02	1.76	0.036	
**PC5 + PR status**										
	PC5	quantitative	na		814	0.85	0.75	0.96	7.1E-03	1.6E-04
	PR status	categorical	positive	negative		1.58	1.23	2.02	2.9E-04	
**PC5 + Her2-status**										
	PC5	quantitative	na		812	0.85	0.76	0.96	0.011	5.7E-04
	Her2 status	categorical	negative	positive		1.67	1.23	2.26	1.0E-03	
**PC5 + Ki-67 status**										
	PC5	quantitative	na		799	0.89	0.79	1.00	0.055	2.7E-03
	Ki-67 status	categorical	<14%	≥14%		1.42	1.11	1.81	5.1E-03	
**PC5, adjusted for all clinical-pathologic characteristics**										
	PC5	quantitative	na			0.84	0.74	0.95	5.9E-03	4.5E-08
	Age at dx	categorical	onset ≥50	onset<50		1.07	0.84	1.36	0.58	
	Nodal status	categorical	1 - 3	4+		1.43	1.12	1.83	4.4E-03	
	Histologic Grade	categorical	G1	G2		1.60	1.00	2.54	0.050	
				G3		1.59	0.98	2.58	0.059	
				GX		1.08	0.57	2.05	0.82	
	Tumor size	categorical	T1	T2		1.38	1.06	1.80	0.017	
				T3		2.24	1.39	3.64	1.0E-03	
	ER status	categorical	positive	negative		0.90	0.63	1.29	0.57	
	PR status	categorical	positive	negative		1.42	1.03	1.98	0.035	
	Her2 status	categorical	negative	positive		1.32	0.95	1.83	0.093	
	Ki-67 status	categorical	<14%	≥14%		1.11	0.85	1.45	0.45	

TABLE S4

MULTIVARIABLE COX PROPORTIONAL HAZARDS FOR INTERACTION OF CLASSIC RISK FACTORS WITH TREATMENT										
**Model**	**Variables**	**Type**	**Reference**	**Tested**	**n**	**HR**	**95% CI**		**p**	**Interaction p**
**CLINICAL-PATHOLOGICAL CHARACTERISTICS**										
**TREATMENT ARM + Age at dx**										
	Treatment Arm * Age at dx	INTERACTION				1.29	0.81	2.07		0.29
	Treatment Arm	categorical	FEC	FEC-P	819	1.01	0.74	1.37	0.015	
	Age at dx	categorical	onset ≥50	onset<50		1.29	0.81	2.07	0.97	
**TREATMENT ARM + Nodal status**										
	Treatment Arm * Nodal status	INTERACTION				0.68	0.43	1.09		0.11
	Treatment Arm	categorical	FEC	FEC-P	819	0.89	0.64	1.23	0.48	
	Nodal status	categorical	1 - 3	4+		2.05	1.50	2.80	6.3E-06	
**TREATMENT ARM + Histologic Grade**										
	Treatment Arm * G2	INTERACTION				2.30	0.84	6.30		0.104
	Treatment Arm * G3	INTERACTION				2.40	0.88	6.54		0.087
	Treatment Arm * GX	INTERACTION				1.72	0.45	6.63		0.428
	Treatment Arm	categorical	FEC	FEC-P	819	0.35	0.14	0.89	0.027	
	Histologic Grade	categorical	G1	G2		1.45	0.84	2.50	0.187	
				G3		1.72	1.00	2.97	0.050	
				GX		1.25	0.57	2.75	0.581	
**TREATMENT ARM + Tumor Size**										
	Treatment Arm * T2	INTERACTION				0.92	0.55	1.53		0.74
	Treatment Arm * T3	INTERACTION				2.33	0.94	5.80		0.07
	Treatment Arm	categorical	FEC	FEC-P	819	0.76	0.50	1.15	0.20	
	Tumor size	categorical	T1	T2		1.70	1.20	2.40	0.0027	
				T3		1.68	0.85	3.33	0.14	
**TREATMENT ARM + ER status**										
	Treatment Arm * ER status	INTERACTION				1.15	0.66	2.00		0.61
	Treatment Arm	categorical	FEC	FEC-P	815	0.74	0.56	0.96	0.026	
	ER status	categorical	positive	negative		1.23	0.86	1.77	0.26	
**TREATMENT ARM + PR status**										
	Treatment Arm * PR status	INTERACTION				0.98	0.59	1.60		0.92
	Treatment Arm	categorical	FEC	FEC-P	814	0.78	0.59	1.05	0.10	
	PR status	categorical	positive	negative		1.46	1.06	2.01	0.019	
**TREATMENT ARM + Her2 status**										
	Treatment Arm * Her2 status	INTERACTION				0.74	0.41	1.36		0.34
	Treatment Arm	categorical	FEC	FEC-P	812	0.77	0.59	1.00	0.047	
	Her2 status	categorical	negative	positive		1.93	1.26	2.96	0.0026	
**TREATMENT ARM + Ki-67 status**										
	Treatment Arm * Ki-67 status	INTERACTION				1.23	0.75	2.02		0.41
	Treatment Arm	categorical	FEC	FEC-P	799	0.71	0.53	0.96	0.025	
	Ki-67 status	categorical	<14%	≥14%		1.29	0.93	1.78	0.13	
**INTRINSIC SUBTYPES**										
**TREATMENT ARM + Intrinsic Subtype**										
	Treatment Arm * Luminal B	Subtype				0.98	0.53	1.81		0.94
	Treatment Arm * Her2-enriched	Subtype				1.08	0.56	2.07		0.82
	Treatment Arm * Basal-like	Subtype				0.82	0.33	2.07		0.68
	Treatment Arm * Normal-like	Subtype				0.48	0.11	2.15		0.33
	Treatment Arm	categorical	FEC	FEC-P	819	0.79	0.49	1.28	0.34	
	Subtype	categorical	Luminal A	Luminal B		1.80	1.19	2.73	0.0055	
				Her2-enriched		2.12	1.34	3.35	0.0014	
				Basal-like		2.15	1.24	3.73	0.006	
				Normal-like		1.47	0.58	3.76	0.42	

TABLE S5

CLINICAL-PATHOLOGICAL CHARACTERISTICS BY QUARTILES OF PC3 & PC4										
Variable	All		PC3				PC4			
			Q1-Q3		Q4		Q1-Q3		Q4	
	n	%	n	%	n	%	n	%	n	%
All	819		614		205		614		205	
**Age (years)**										
<50	394	48.1%	281	45.8%	113	55.1%	294	47.9%	100	48.8%
≥ 50	425	51.9%	333	54.2%	92	44.9%	320	52.1%	105	51.2%
**Nodal Status**										
1-3	506	61.8%	375	61.1%	131	63.9%	393	64.0%	113	55.1%
>3	313	38.2%	239	38.9%	74	36.1%	221	36.0%	92	44.9%
**Histologic Grade**										
G1	108	13.2%	74	12.1%	34	16.6%	92	15.0%	16	7.8%
G2	337	41.1%	236	38.4%	101	49.3%	253	41.2%	84	41.0%
G3	315	38.5%	256	41.7%	59	28.8%	221	36.0%	94	45.9%
GX	59	7.2%	48	7.8%	11	5.4%	48	7.8%	11	5.4%
**Primary Tumor Size**										
T1	342	41.8%	246	40.1%	96	46.8%	262	42.7%	80	39.0%
T2	431	52.6%	337	54.9%	94	45.9%	324	52.8%	107	52.2%
T3	46	5.6%	31	5.0%	15	7.3%	28	4.6%	18	8.8%
**Estrogen receptor (IHC)**										
Negative	172	21.0%	156	25.4%	16	7.8%	108	17.6%	64	31.2%
Positive	643	78.5%	455	74.1%	188	91.7%	503	81.9%	140	68.3%
**Progesterone receptor (IHC)**										
Negative	246	30.0%	216	35.2%	30	14.6%	152	24.8%	94	45.9%
Positive	568	69.4%	394	64.2%	174	84.9%	458	74.6%	110	53.7%
**Her2 status (CISH)**										
Negative	696	85.0%	511	83.2%	185	90.2%	586	95.4%	110	53.7%
Positive	116	14.2%	98	16.0%	18	8.8%	22	3.6%	94	45.9%
**Ki-67 (IHC)**										
Low (<14%)	557	68.0%	407	66.3%	150	73.2%	413	67.3%	144	70.2%
High (≥14%)	242	29.5%	192	31.3%	50	24.4%	183	29.8%	59	28.8%
**PAM50 Intrinsic subtypes**										
Luminal A	280	34.2%	175	28.5%	105	51.2%	253	41.2%	27	13.2%
Luminal B	261	31.9%	213	34.7%	48	23.4%	206	33.6%	55	26.8%
Her2-enriched	175	21.4%	132	21.5%	43	21.0%	54	8.8%	121	59.0%
Basal-like	71	8.7%	68	11.1%	3	1.5%	71	11.6%	0	0.0%
Normal-like	32	3.9%	26	4.2%	6	2.9%	30	4.9%	2	1.0%

Color key

Color	Meaning
Yellow	>=5% absolute difference compared to percentage in the overal dataset
Orange	<5% absolute difference compared to percentage in the overal dataset

TABLE S6

INTRINSIC DIMENSIONS, DESCRIPTIVES by OTHER TUMOR CHARACTERISTICS										
Variable	PC1		PC2		PC3		PC4		PC5	
	mean	sd	mean	sd	mean	sd	mean	sd	mean	sd
**All**	**0.0**	5.4	**0.0**	5.3	**0.0**	3.6	**0.0**	2.8	**0.0**	2.2
**Age at dx**										
<50	**0.1**	5.4	**0.3**	5.2	**0.3**	3.5	**0.1**	2.9	**0.0**	2.2
≥ 50	**0.0**	5.3	**-0.3**	5.3	**-0.2**	3.6	**0.0**	2.8	**0.1**	2.2
**Nodal Status**										
1-3	**-0.3**	5.7	**0.4**	5.4	**0.1**	3.6	**-0.1**	2.7	**0.0**	2.2
>3	**0.5**	4.8	**-0.6**	4.9	**0.0**	3.5	**0.2**	3.1	**0.1**	2.2
**Histologic grade**										
G1	**-3.8**	3.6	**0.9**	5.1	**0.9**	3.0	**-0.3**	1.7	**0.2**	1.9
G2	**-1.5**	4.2	**-0.6**	4.9	**0.4**	3.8	**0.1**	2.7	**0.0**	2.3
G3	**3.3**	5.2	**0.1**	5.7	**-0.7**	3.5	**0.1**	3.3	**-0.1**	2.2
GX	**-2.3**	5.4	**1.1**	5.0	**0.3**	2.9	**-0.1**	2.3	**0.2**	1.8
**Tumor Size**										
T1	**-1.0**	5.1	**0.3**	5.1	**0.3**	3.6	**0.0**	2.8	**0.0**	2.2
T2	**0.6**	5.4	**-0.4**	5.4	**-0.2**	3.6	**-0.1**	2.8	**0.0**	2.2
T3	**1.5**	5.8	**0.9**	5.0	**0.6**	3.1	**0.6**	3.3	**0.7**	2.4
**Estrogen receptor status (IHC)**										
Negative	**5.5**	5.9	**2.9**	5.2	**-2.0**	3.5	**0.6**	4.1	**0.1**	2.2
Positive	**-1.5**	4.1	**-0.8**	5.0	**0.6**	3.4	**-0.1**	2.4	**0.0**	2.2
**Progesterone receptor status (IHC)**										
Negative	**4.4**	5.3	**1.3**	5.7	**-1.5**	3.6	**0.9**	3.5	**0.5**	2.2
Positive	**-1.9**	4.1	**-0.6**	5.0	**0.7**	3.4	**-0.3**	2.4	**-0.2**	2.2
**Her2 status (FISH)**										
Negative	**-0.5**	5.5	**0.2**	5.3	**0.3**	3.5	**-0.6**	2.3	**-0.1**	2.2
Positive	**2.9**	3.3	**-1.2**	5.0	**-1.6**	3.6	**3.6**	3.0	**0.7**	2.0
**Ki-67 status (IHC)**										
Low (<14%)	**-1.4**	4.7	**0.0**	4.9	**0.4**	3.5	**0.2**	2.7	**0.1**	2.2
High (≥14%)	**3.4**	5.3	**-0.1**	5.9	**-0.7**	3.6	**-0.3**	3.3	**-0.2**	2.2
**Subtype Prediction**										
Luminal A	**-4.3**	3.1	**1.2**	3.3	**1.4**	3.1	**-0.5**	1.9	**-0.5**	1.9
Luminal B	**0.3**	3.0	**-5.3**	3.1	**-0.4**	3.5	**-0.5**	2.4	**0.1**	2.4
Her2-enriched	**2.4**	3.2	**1.5**	3.3	**-0.8**	4.1	**2.8**	2.9	**0.5**	2.3
Basal-like	**11.4**	2.2	**6.6**	3.0	**-1.6**	2.3	**-2.8**	2.3	**-0.1**	2.0
Normal-like	**-3.7**	4.1	**10.0**	2.6	**0.0**	3.2	**-0.6**	1.8	**1.4**	2.0

Color key

(yellow)	mean value >2.0
(orange)	mean value <-2.0
