# Supplementary material for: Re-interpretation of PAM50 gene expression as quantitative tumor dimensions shows utility for clinical trials: application to prognosis and response to paclitaxel in breast cancer
Source: Breast Cancer Res Treat. 2019 Jan 23;175(1):129–39. doi: 10.1007/s10549-018-05097-5 (PMC6491406; doi:10.1007/s10549-018-05097-5)
Supplement: Supplementary file 2 — Supplementary material 2 (DOCX 13 KB) [file 10549_2018_5097_MOESM2_ESM.docx]

**Supplemental Figure legends**

**Figure S1.** Graphs indicating the quantitative dimensions in the LACE/Pathways data and the GEICAM study. For comparison each tumor is colored based on their intrinsic PAM50 subtype (red=Basal-Like; pink=HER2-Enriched; light blue=Luminal B; dark blue=Luminal A; green=Normal-Like). **A.** 3D plot of PC1-PC2-PC4 for the LACE/Pathways data[1]. **B**. 3D plot of PC1-PC2-PC4 for the GEICAM data (this study). **C**. PC3 and PC5 distributions for the LACE/Pathways data, split based on intrinsic PAM50 subtype, indicting little to no association. **D**. PC3 and PC5 distributions for the GEICAM data, split based on intrinsic PAM50 subtype, indicting little to no association.

**Figure S2**. Disease-free survival by treatment arm. Number at risk for each group are shown below the plot.

**Figure S3**. Disease-free survival by treatment arm and PC3 quartiles (Q1-Q3, Q4). Kaplan-Meier curves for the following four groups: low PC3 and FEC plus paclitaxel treatment (“Q1-3.FEC-P”); low PC3 and FEC treatment only (“Q1-3.FEC”); high PC3 and FEC plus paclitaxel treatment (“Q4.FEC-P”); high PC3 and FEC treatment only (“Q4.FEC”). Number at risk for each group are shown below the plot.

**Figure S4**. Disease-free survival by treatment arm and PC4 quartiles (Q1-Q3, Q4). Kaplan-Meier curves for the following four groups: low PC4 and FEC plus paclitaxel treatment (“Q1-3.FEC-P”); low PC4 and FEC treatment only (“Q1-3.FEC”); high PC4 and FEC plus paclitaxel treatment (“Q4.FEC-P”); high PC4 and FEC treatment only (“Q4.FEC”). Number at risk for each group are shown below the plot.

**Figure S5**. Box and whisker plots for dimensions PC1 – PC5 by clinical -pathological characteristics.

**Supplemental Table headers**

**Table S1.** Gene coefficients for PC1 – PC5, as previously derived from the LACE/PW data[1].

**Table S2**. Characteristics of dimensions PC1 – PC5 by trial arm.

**Table S3**. Multivariable proportional hazards for PC5 for disease-free survival.

Hazard ratios for categorical variables are comparisons to the reference category, as noted. Hazard ratios for quantitative traits are per standard deviation (SD) unit for the trait.

**Table S4**. Multivariable Cox proportional hazards for interaction of classic risk factors with treatment.

**Table S5.** Clinical-pathological characteristics by PC3 and PC4 quartiles (Q1-Q3, Q4).

Yellow-shaded cells indicate an *increase* of 5 or more percentage points from the overall dataset. Brown-shaded cells indicate a *decrease* of 5 or more percentage points from the overall dataset.

**Table S6**. Mean and standard deviation for dimensions PC1 – PC5 by clinical -pathological characteristics.
